# Alpha thalassemia and alpha-MRE haplotypes in Uruguayan patients with microcytosis and hypochromia without anemia

**DOI:** 10.1590/1678-4685-GMB-2020-0399

**Published:** 2021-03-26

**Authors:** Ana María Soler, Bruna Facanali Piellusch, Lorena da Silveira, Gisele Audrei Pedroso, Pablo López, Enrique Savio, María de Fatima Sonati, Julio da Luz

**Affiliations:** 1 Universidad de la República (UdelaR), Centro Universitario Regional (CENUR) Litoral Norte, Departamento de Ciencias Biológicas, Laboratorio de Genética Molecular Humana, Salto, Uruguay.; 2 Universidade Estadual de Campinas (UNICAMP), Faculdade de Ciências Médicas, Departamento de Patología Clínica, Campinas, SP, Brazil.; 3 Universidad de la República (UdelaR), Facultad de Medicina, Hospital de Clínicas Manuel Quintela, Departamento de Laboratorio de Patología Clínica, Montevideo, Uruguay.; 4 Administración de los Servicios de Salud del Estado (ASSE), Hospital Departamental de Salto, Servicio de Laboratorio Clínico, Salto, Uruguay.

**Keywords:** Alpha-thalassemia, alpha-MRE, microcytosis, hypochromia, Uruguay

## Abstract

Alpha thalassemia is the most common genetic disorder across the world, being the α-^3.7^ deletion the most frequent mutation. In order to analyze the spectrum and origin of alpha thalassemia mutations in Uruguay, we obtained a sample of 168 unrelated outpatients with normal hemoglobin levels with microcytosis and hypochromia from two cities: Montevideo and Salto. The presence of α-thalassemia mutations was investigated by gap-PCR, restriction endonucleases analysis and HBA2 and HBA1 genes sequencing, whereas the alpha-MRE haplotypes were investigated by sequencing. We found 55 individuals (32.7%) with α-thalassemia mutations, 51(30.4%) carrying the -α^3.7^ deletion, one with the -α^4.2^ deletion and three having the rare punctual mutation HBA2:c.-59C>T. Regarding alpha-MRE analysis, we observed a significant higher frequency of haplotype D, characteristic of African populations, in the sample with the -α^3.7^ deletion. These results show that α-thalassemia mutations are an important determinant of microcytosis and hypochromia in Uruguayan patients with microcytosis and hypochromia without anemia, mainly due to the -α^3.7^ deletion. The alpha-MRE haplotypes and the α-thalassemia mutations spectrum suggest a predominant, but not exclusive, African origin of these mutations in Uruguay.

## Introduction

α-thalassemia is the most common genetic disorder across the world. It is mainly caused by deletions of one (-α) or both (--) HBA genes of the α-globin gene cluster, although small deletions or point mutations also contribute to the α-thalassemia mutations spectrum (Foglietta et al., 1996; Huisman et al., 1998; Steinberg et al., 2001).

The absence (a^0^-thalassemia) or reduction (a^+^-thalassemia) of α-globin synthesis produces an imbalance between a and b-globin chains. This imbalance results in a deficient synthesis of hemoglobin in erythroid cells, causing microcytosis (reduction of mean corpuscular volume, MCV) and hypochromia (reduction of mean corpuscular hemoglobin, MCH). Microcytosis and hypochromia with normal levels of HbA2 can also be due to iron deficiency or anemia of chronic disease (Weatherall and Clegg, 1981; Weiss and Goodnough, 2005).

Hematological alterations depend on the number of α-globin genes affected (Steinberg et al., 2001). They range from almost asymptomatic or mild microcytic anemia in individuals with one or two affected genes to life incompatibility in the case of the hydrops fetalis syndrome due to the loss of the four α-globin genes. The consequence of losing or carrying alterations in three genes is the presence of a hemolytic anemia with marked variation in phenotypic expression, characterized by the presence of H hemoglobin (b4) (Higgs et al., 1989).

α-thalassemia reaches high frequencies in regions where malaria is or has been endemic, as in African, Mediterranean basin and Southeast Asian populations (Weatherall and Clegg, 2001). Furthermore, α-thalassemia mutations have spread across the world by immigration and slave trade (Isola, 1975; Sans, 1994; Weatherall and Clegg, 2001).

The a^+^-thalassemia due to a deletion of 3.7Kb (-α^3.7^ deletion) is the most common cause of α-thalassemia, which affects both HBA1 and HBA2 genes, resulting in a single hybrid gene (HBA2-HBA1). Another frequent cause of a^+^-thalassemia is the deletion of 4.2Kb (-α^4.2^ deletion) which deletes the entire HBA2 gene (Higgs et al., 1989; Foglietta et al., 1996). African and some Asian populations present the highest frequencies of -α^3.7^ deletion, while the -α^4.2^ deletion is more frequent in Asian populations (Kattamis et al., 1996; Weatherall and Clegg, 2001). Both deletions are also observed in European and Mediterranean populations at variable frequencies. For example, in Sardinian and Cypriot populations the -α^3.7^ deletion frequency is 12.6% and 14.0% respectively, whereas in the Basque population it is practically inexistent (Steinberg et al., 2001; Weatherall and Clegg, 2001).

The loss of the two α-globin genes in *cis* results in a^0^-thalassaemias. In the Mediterranean region, the --^MED^ and --^20.5^ deletions are the most frequent mutations, whereas in African populations they are almost absent (Kattamis et al., 1996; Steinberg et al., 2001; Weatherall and Clegg, 2001). On the other hand, the deletion of a pentanucleotide (TGAGG) located at the 5’ end of the HBA2 gene IVS-I (a^HpHI^a), a point mutation at the initiation codon (ATG-ACG) of the HBA2 gene (α^NcoI^α), as well as a point mutation (ATG-GTG) in the HBA1 gene (αα^NcoI^), are the most common non-deletional mutations of α-thalassemia in the Mediterranean region (Higgs et al., 1989; Foglietta et al., 1996; Kattamis *et al*., 1996; Steinberg *et al*., 2001).

For a pediatric Uruguayan population, da Luz et al. (2013) previously reported that the estimated incidence of α-thalassemia was 3.3% and that only the -α^3.7^ deletion was observed. The --^20.5^ mutation was observed only in one individual from an enriched sample with microcytosis and hypochromia without iron deficiency and normal levels of HbA2. Interestingly, the -α^3.7^ deletion was observed mainly in Afro-descendants, classification based on their ancestor’s origin (da Luz *et al*., 2013). Soler et al. (2016) reported for the first time in Latin America the -α^5.2^ deletion, a mutation observed in Greek and Italian populations (Pressley et al., 1980; Fortina et al., 1994).

Previous studies, have shown that the Uruguayan population is tri-hybrid, with a greater genetic contribution of European populations (~84%, mainly Spaniards and Italians) followed in smaller proportions by Native Americans (~10%) and sub-Saharan populations (~6%) (Sans, 1994; Hidalgo et al., 2005; Sans *et al*., 2006).

In order to contribute to knowledge of α-thalassemia mutations in the Uruguayan population, we analyzed 168 outpatients from Montevideo and Salto (117 and 51 respectively) who showed normal Hb levels and reduced levels of VCM and MCH. Additionally, to investigate the -α^3.7^ deletion origin, we sequenced the alpha-MRE region in individuals with and without this mutation.

## Subjects and Methods

A total of 168 unrelated individuals among 1 and 84 years old were analyzed, 117 outpatients from Hospital de Clínicas - Universidad de la República (UdelaR), Montevideo and 51 from Hospital Regional Salto - Administración de Servicios de Salud del Estado (ASSE), recruited in 2015. The protocol and procedure employed in this research were in compliance with the Helsinki Declaration. Each subject and the children’s parents gave written informed consent to participate in the study.

All individuals present normal levels of Hb and diminished levels of VCM and MCH. In adults (≥15 years old) the Hb levels were ≥ 13 g/dL for men and ≥ 12 g/dL for women. For both sexes, the VCM and MCH levels were ≤ 80 fl and ≤ 27 pg respectively. For individuals between 12 and 15 years old, we considered Hb levels ≥ 12 and VCM and MCH levels ≤ 78 fl and ≤ 26 pg respectively. In children under 12 years old, the levels of Hb were greater than 11 g/dL. In the range between ≥ 5 and < 12 years old the VCM and MCH levels were ≤ 77 fl and ≤ 25 pg respectively, whereas for children under 5 years old VCM and MCH levels were ≤ 75 fl and ≤ 24 pg respectively. The Hb thresholds were according those established by World Health Organization (de Benoist et al., 2008) and the VCM and MCH levels according to Besses et al. (2007).

Genomic DNA was extracted from venous peripheral blood by the salting out method (Miller et al., 1988). All individuals were analyzed for the presence of deletional and non-deletional α-thalassemia mutations. The seven most common deletional α-thalassemia (-α^3.7^, -α^4.2^, --^SEA^, --^FIL^, --^MED^, -α^20.5^, and --^THAI^) were checked by multiplex gap polymerase chain reaction (gap-PCR) and agarose gel electrophoresis, according to conditions already described (Tan et al., 2001). The most common non-deletional alpha thalassemia mutations were analyzed by restriction fragment length polymorphism (RFLP) from products amplified by PCR (α^Hph^α, α^NcoI^α and αα^NcoI^) (Hall et al., 1993). Additionally, HBA1 and HBA2 genes were sequenced to analyzes the presence of point mutations or small deletion (Pedroso et al., 2018; Dodé et al., 1990).

The α-MRE haplotypes were determined in 157 individuals: 78 individuals with the -α^3.7^ deletion and 79 without α-thalassemia mutations. A 310bp DNA fragment was amplified and sequenced according to conditions previously described (Harteveld *et al*., 2002). Haplotypes were constructed by assuming that the presence of two common haplotypes was more probable than the combination of one common and one rare haplotype or two rare haplotypes (Long et al., 1990; Castro de Guerra et al., 1997).

### Statistical methods

The genotypic and allelic frequencies were estimated by gene counting. The Hardy-Weinberg equilibrium, the estimation of heterogeneity among the samples by the exact test of population differentiation, and the pairwise F_ST_ were evaluated using Arlequin software package v 3.5.2.2 (Excoffier and Lischer, 2010). F_ST_ distances between Uruguayan population and other populations were represented in two dimensions by multidimensional scaling (MDS) using the software SPSS 22.0 Data of other populations were obtained from Harteveld *et al*. (2002), Ribeiro et al. (2003) and from the 1000 Genomes Database.

## Results

α-thalassemia mutations were found in 55 individuals (32.7%). The distribution and frequencies of α-thalassemia mutations are showed in Table 1. The -α^3.7^ deletion was the most frequent mutation observed, 49 individuals heterozygous -α^3.7^/αα and two homozygous -α^3.7^/-α^3.7^, whereas the -α^4.2^ deletion was observed in only one individual. Three individuals were heterozygous for the point mutation HBA2: c.-59C>T. The mutations distribution and frequencies do not differ significantly between the two cities (Montevideo and Salto) analyzed.

**Table 1 -:** Alpha thalassaemia mutations found in the Uruguayan microcytic and hypochromic sample.

Genotype	Montevideo	Salto	Total
	N (%)	N (%)	N (%)
αα/αα	77 (65.0)	36 (70.7)	113 (67.3)
-α^3.7^/αα	36 (30.8)	13 (25.5)	49 (29.2)
-α^3.7^/-α^3.7^	1 (0.9)	1 (1.9)	2 (1.2)
-a^4.2^/αα	1 (0.9)		1 (0.6)
HBA2:c.-59C>T	2 (1.7)	1 (1.9)	3 (1.7)
Total	117	51	168

The number of different α-MRE genotypes and α-MRE haplotypes are showed in Table 2 and Table S1. The distribution of α-MRE genotypes and α-MRE haplotypes were significantly different between individuals with and without the -α^3.7^ deletion (*p*<0.05).

**Table 2 -:** α-MRE genotypes in Uruguayan population with and without -a^3.7^ deletion.

Population	a-MRE genotypes						N	*p*
	AA (%)	AB (%)	AD (%)	BB (%)	BD (%)	DD (%)		
a^3.7^ deletion	23 (29.5)	31 (39.7)	10 (12.8)	5 (6.4)	8(10.3)	1 (1.3)	78	*0.0053*
aa/aa	32 (40.5)	33 (41.8)	1 (1.3)	11 (13.9)	2 (2.5)		79	

**Figure 1 -:**
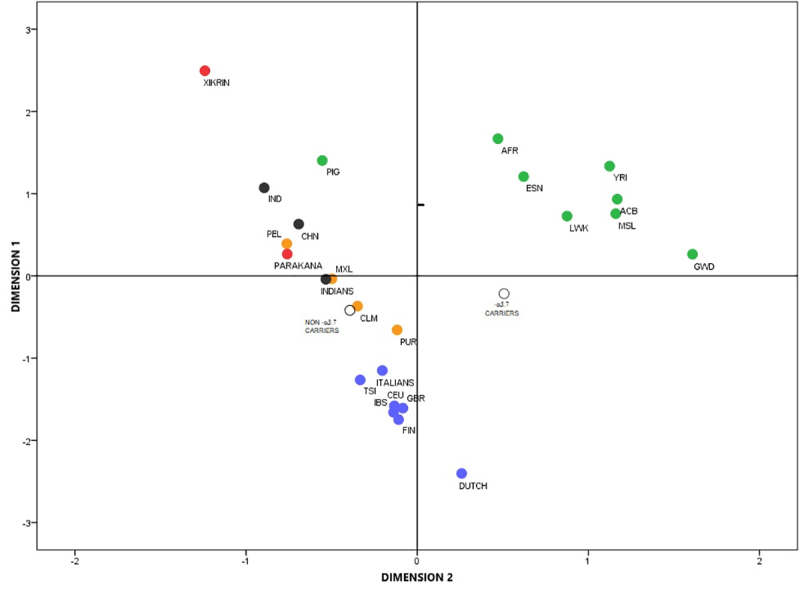
Multidimensional scaling (MDS) based on pairwise F_ST_ genetic distances calculated from alpha-MRE genotypes.

Multidimensional scaling (MDS) showed the subsample without -α^3.7^ deletion grouped with other Latino-American populations whereas, the sample with the -α^3.7^ deletion was split from these in the second dimension and was found more related to African populations (Figure 1). The lower genetic distances from the subsample without the -α^3.7^ deletion were with Latin-American populations from Peru, Colombia, Puerto Rico and Mexico. However, the subsample with the -α^3.7^ deletion presented the lowest genetic distances with Africans and Afro-Latin-American populations (Table S2).

Stress: 0.01390; RSQ: 0.99913. -α3.7 CARRIERS & NON -α3.7 CARRIERS: Uruguayan populations. Green: African and African derived populations. YRI: Yoruba in Ibadan, Nigeria. ACB: African Caribbeans in Barbados. GWD: Gambian in Western Divisions in the Gambia. MSL: Mende in Sierra Leone. ESN: Esan in Nigeria. LWK: Luhya in Webuye, Kenya. AFR: Bantu-speaking Africans. PIG: Pygmies from the Central African Republic. Orange: Admixed Latin-American populations. PEL: Peruvians from Lima, Peru. PUR: Puerto Ricans from Puerto Rico. CLM: Colombians from Medellin, Colombia. MXL: Mexican Ancestry from Los Angeles USA. Blue: European populations. IBS: Iberian Population in Spain. TSI: Toscani in Italia. CEU: Northern and Western European Ancestry. FIN: Finnish in Finland. ITALIANS. DUTCH. GBR: British in England and Scotland. Black: Asian populations. IND: Indonesians from Java. CHN: Southern Chinese. INDIANS: Andra-Pradesh, India. (1000genomes; Harteveld *et al*., 2002; Ribeiro et al., 2003).

## Discussion

Our results show that α-thalassemia mutations explain an important percentage (32.7%) of the microcytosis and hypochromia observed in the Uruguayan population without anemia. Furthermore, the -α^3.7^ deletion was the most frequent mutation. The only non-deletional α-thalassemia mutation observed (HBA2 c.-59C>T), affects the last nucleotide of TATA box. This mutation was reported only once in an individual from Australia. In vitro analysis shows a reduction of the transcriptional activity in 53.7% when compared with the normal allele (HBA2 wild type) (Qadah et al., 2014). In our study, this mutation was observed in three non-related individuals from two different regions of Uruguay. This data suggests a possible founder effect in the Uruguayan population.

The α-thalassemia observed frequency (32.7%) is similar to previously reported for Rio Grande do Sul, Brazil (31.7%) (Wagner et al., 2010). However, it is lower than the one reported for Campinas, Southeastern Brazil (49.9%) (Borges et al., 2001). The high frequency reported for Campinas could be explained by a greater African contribution in this population. Nevertheless, in the Campinas sub-sample self-reported as Caucasian, the α-thalassemia frequency was also higher (41.5%) than the observed for Rio Grande do Sul and Uruguay (Borges *et al*., 2001; Wagner *et al*., 2010; da Luz et al., 2013). We hypothesize that those differences may be due to African genetic ancestry not detected in this sub-sample. Several studies had showed that Latin-American self-reported as whites present variable levels of African and/or Native American genetic ancestry. For example, in a female population from Brasilia, the percentage of African ancestry in self-reported whites was 17.2% (Lins et al., 2011). Another explanation may be that the Campinas population self-reported as white presents a greater European contribution from populations with higher α-thalassemia frequencies, as South-Italians (Weatherall and Clegg, 2001).

The observed similarity between Rio Grande do Sul and Uruguay is according with the common historical origin from these populations as well as with genetic data showing a high similarity at DNA mitochondrial level (Marrero et al., 2007).

In relation to alpha-MRE haplotypes, the observed differences between the two Uruguayan subsamples (with and without 3.7 deletion) is due to a high frequency of haplotype D in α-thalassemia subsample (Table S1). Haplotype D is characteristic of African populations. It is practically absent in others populations and therefore, is a useful marker for African ancestry (Harteveld *et al*., 2002). Moreover, the haplotype B frequency for both Uruguayan subsamples was lower than the ones observed for European populations and similar to observed for other admixed Latin-American and Asian populations (Harteveld *et al*., 2002; Ribeiro et al., 2003). With exception of Pigmies populations, the MDS plot of F_ST_ genetic distances calculated from alpha-MRE haplotypes clearly discriminated among African, European and Asian populations. As expected, Native American populations were grouped with Asian populations. Furthermore, admixed Latin-American populations were placed at an intermediate position between Asian and European populations according to admixture degree. For example, Peruvian and Mexican populations, who have the greater Native American ancestry, were clustered with Asian populations, whereas Colombian and Puerto Rico populations were placed near European populations. Moreover, the Puerto Rico population, which has the greater African ancestry, was displaced towards the African cluster (Rishishwar et al., 2015; Norris et al., 2018). The position of the Uruguayan subsample without the -α^3.7^ deletion next to the Colombian population, agrees with previous reports about the genetic ancestry of these two populations. Similar to reported for a Colombian population from Medellin (Norris *et al*., 2018), the Uruguayan population shows a greater European genetic ancestry (76.2%-84%) followed in smaller proportions by Native Americans (10% - 14.7%) and African ancestry (6% - 9.1%) (Sans, 1994; Hidalgo et al., 2005; Sans *et al*., 2006; Bonilla et al., 2015). However, the Uruguayan α-thalassemia subsample is placed near African populations, suggesting a greater African ancestry in this subsample. This is in accordance with previous studies that had shown a greater African ancestry based on self-perception in individuals with the -α^3.7^ deletion (da Luz et al., 2013).

In summary, α-thalassemia mutations explain a significant proportion of microcytosis and hypochromia in the Uruguayan population. The alpha-MRE haplotypes and the α-thalassemia mutations spectrum observed suggest a predominant, but not exclusive, African origin of the -α^3.7^ deletion in Uruguay.

## Supplementary material

The following online material is available for this article:

Table S1 - α-MRE haplotypes.

Table S2 - F_ST_ genetic distances.
