# Supplementary material for: Alpha thalassemia and alpha-MRE haplotypes in Uruguayan patients with microcytosis and hypochromia without anemia
Source: Genet Mol Biol. 2021 Mar 26;44(2):e20200399. doi: 10.1590/1678-4685-GMB-2020-0399 (PMC7995682; doi:10.1590/1678-4685-GMB-2020-0399)
Supplement: Table S1 - α-MRE haplotypes. [file 1415-4757-GMB-44-2-e20200399-s1.pdf]

Genetics and Molecular Biology

https://doi.org/10.1590/1678-4685-GMB-2020-0399

Supplementary Material to "Alpha thalassemia and alpha-MRE haplotypes in Uruguayan patients with microcytosis and hypochromia without anemia"

Table S1. α-MRE haplotypes in Uruguayan and other populations.

	Haplotypes						
	A (%)	B (%)	C (%)	D (%)	E (%)	F (%)	N
UY $\alpha^{3.7}$	87 (55.8)	49 (31.4)		20 (12.8)			156
UY αα	98 (62.0)	57 (36.1)		3 (1.9)			158
YRI*	139 (64.4)	25 (11.6)		48 (22.2)	4 (1.8)		216
ACB*	116 (60.4)	30 (15.6)	1 (0.5)	43 (22.4)	2 (1.1)		192
GWD*	113 (50.0)	48 (21.2)		60 (26.6)	5 (2.2)		226
MSL*	101 (59.4)	31 (18.2)		38 (22.4)			170
ESN*	132 (66.7)	30 (15.1)		33 (16.7)	3 (1.5)		198
LWK*	194 (60.6)	63 (19.7)		63 (19.7)			320
PEL*	119 (70.0)	50 (29.4)		1 (0.6)			170
PUR*	119 (57.2)	79 (38.0)	2 (1.0)	8 (3.8)			208
CLM*	116 (61.7)	67 (35.6)		5 (2.7)			188
MXL*	84 (65.6)	41 (32.0)	1 (0.8)	2 (1.6)			128
IBS*	108 (50.5)	105 (49.0)	1 (0.5)				214
TSI*	117 (54.7)	97 (45.3)					214
CEU*	101 (51.0)	95 (48.0)	2 (1.0)				198
FIN*	99 (50.0)	99 (50.0)					198
GBR*	92 (50.5)	88 (48.4)	2 (1.1)				182
DUTCH**	30 (43.0)	40 (57.0)					70
ITALIANS**	59 (55.7)	46 (43.4)	1 (0.9)				106
INDIANS**	52 (66.7)	25 (32.1)	1 (1.2)				78
IND**	53 (78.0)	15 (22.0)					68
CHN**	34 (73.9)	12 (26.1)					46
AFR**	47 (73.0)	7 (11.0)		10 (16.0)			64
PIG**	91 (75.8)	19 (15.8)		6 (5.0)	2 (1.7)	2 (1.7)	120
PARAKANA***	98 (70.0)	42 (30.0)					140
XIKRIN***	166 (87.4)	24 (12.6)					190

*1000genomes. **Harteveld et al., 2002. ***Ribeiro et al., 2003. UY: Uruguayan population. YRI: Yoruba in Ibadan, Nigeria. ACB: African Caribbeans in Barbados. GWD: Gambian in Western Divisions in the Gambia. MSL: Mende in Sierra Leone. ESN: Esan in Nigeria. LWK: Luhya in Webuye, Kenya. PEL: Peruvians from Lima, Peru. PUR: Puerto Ricans from Puerto Rico. CLM: Colombians from Medellin, Colombia. MXL: Mexican Ancestry from Los Angeles USA. IBS: Iberian Population in Spain. TSI: Toscani in Italia. CEU: Northern and Western European Ancestry. FIN: Finnish in Finland. GBR: British in England and Scotland. IND: Indonesians from Java. CHN: Southern Chinese. AFR: Bantu-speaking Africans. PIG: Pygmies from the Central African Republic.
