# Supplementary material for: Alpha thalassemia and alpha-MRE haplotypes in Uruguayan patients with microcytosis and hypochromia without anemia
Source: Genet Mol Biol. 2021 Mar 26;44(2):e20200399. doi: 10.1590/1678-4685-GMB-2020-0399 (PMC7995682; doi:10.1590/1678-4685-GMB-2020-0399)
Supplement: Table S2 - FST genetic distances. [file 1415-4757-GMB-44-2-e20200399-s2.pdf]

Genetics and Molecular Biology

https://doi.org/10.1590/1678-4685-GMB-2020-0399

Supplementary Material to "Alpha thalassemia and alpha-MRE haplotypes in Uruguayan patients with microcytosis and hypochromia without anemia"

Table S2. F_{ST} genetic distances between populations.

	1	2	3	4	5	6	7	8	9	10	11	12	13	14	15	16	17	18	19	20	21	22	23	24	25	26
1-UY $\alpha^{3.7}$	0.000																									
2-UY αα	0.010	0.000																								
3-YRI	0.044	0.087	0.000																							
4-ACB	0.025	0.069	0.002	0.000																						
5-GWD	0.021	0.074	0.022	0.008	0.000																					
6-MSL	0.018	0.060	0.001	0.005	0.005	0.000																				
7-ESN	0.030	0.059	0.000	0.002	0.030	0.004	0.000																			
8-LWK	0.014	0.048	0.004	0.002	0.010	0.004	0.002	0.000																		
9-PEL	0.031	0.008	0.073	0.066	0.092	0.062	0.042	0.047	0.000																	
10-PUR	0.006	0.002	0.090	0.068	0.064	0.059	0.066	0.048	0.023	0.000																
11-CLM	0.009	0.006	0.083	0.065	0.070	0.057	0.056	0.045	0.008	0.002	0.000															
12-MXL	0.014	0.004	0.072	0.059	0.074	0.053	0.044	0.040	0.003	0.005	0.003	0.000														
13-IBS	0.040	0.025	0.166	0.135	0.111	0.121	0.139	0.109	0.075	0.013	0.026	0.045	0.000													
14-TSI	0.027	0.009	0.141	0.114	0.100	0.102	0.113	0.089	0.049	0.003	0.010	0.024	0.002	0.000												
15-CEU	0.036	0.021	0.158	0.128	0.106	0.115	0.132	0.103	0.069	0.010	0.022	0.039	0.005	0.003	0.000											
16-FIN	0.043	0.028	0.172	0.140	0.114	0.126	0.146	0.114	0.081	0.015	0.029	0.049	0.005	0.001	0.004	0.000										
17-GBR	0.037	0.022	0.161	0.130	0.107	0.117	0.135	0.105	0.072	0.011	0.023	0.042	0.005	0.002	0.005	0.005	0.000									
18-DUTCH	0.074	0.068	0.221	0.182	0.138	0.166	0.199	0.156	0.143	0.045	0.068	0.098	0.003	0.018	0.005	0.001	0.004	0.000								
19-ITALIAN	0.020	0.002	0.127	0.101	0.088	0.089	0.100	0.077	0.039	0.003	0.003	0.015	0.001	0.007	0.003	0.000	0.003	0.023	0.000							
20-INDIANS	0.017	0.005	0.076	0.063	0.078	0.057	0.046	0.044	0.006	0.005	0.004	0.010	0.045	0.024	0.040	0.050	0.042	0.101	0.014	0.000						
21-IND	0.059	0.039	0.067	0.069	0.108	0.069	0.037	0.055	0.002	0.059	0.038	0.018	0.129	0.097	0.122	0.137	0.126	0.216	0.087	0.014	0.000					

	1	2	3	4	5	6	7	8	9	10	11	12	13	14	15	16	17	18	19	20	21	22	23	24	25	26
22-CHN	0.033	0.012	0.062	0.058	0.088	0.056	0.032	0.042	0.012	0.029	0.012	0.003	0.089	0.060	0.082	0.095	0.085	0.162	0.049	0.007	0.014	0.000				
23-AFR	0.055	0.082	0.003	0.012	0.053	0.017	0.004	0.014	0.052	0.092	0.079	0.061	0.178	0.148	0.170	0.186	0.173	0.254	0.135	0.064	0.034	0.037	0.000			
24-PIG	0.061	0.054	0.045	0.051	0.095	0.054	0.021	0.042	0.015	0.072	0.052	0.031	0.151	0.118	0.143	0.159	0.147	0.237	0.107	0.029	0.006	0.002	0.011	0.000		
25-PAR	0.029	0.005	0.077	0.068	0.092	0.064	0.046	0.049	0.006	0.019	0.005	0.005	0.068	0.042	0.062	0.073	0.065	0.133	0.033	0.008	0.005	0.011	0.058	0.019	0.000	
26-XIKRIN	0.161	0.144	0.114	0.132	0.193	0.142	0.084	0.114	0.075	0.167	0.139	0.112	0.262	0.222	0.255	0.274	0.262	0.398	0.227	0.115	0.024	0.054	0.065	0.021	0.084	0.000

UY: Uruguayan population. YRI: Yoruba in Ibadan, Nigeria. ACB: African Caribbeans in Barbados. GWD: Gambian in Western Divisions in the Gambia. MSL: Mende in Sierra Leone. ESN: Esan in Nigeria. LWK: Luhya in Webuye, Kenya. PEL: Peruvians from Lima, Peru. PUR: Puerto Ricans from Puerto Rico. CLM: Colombians from Medellin, Colombia. MXL: Mexican Ancestry from Los Angeles USA. IBS: Iberian Population in Spain. TSI: Toscani in Italia. CEU: Northern and Western European Ancestry. FIN: Finnish in Finland. GBR: British in England and Scotland. IND: Indonesians from Java. CHN: Southern Chinese. AFR: Bantu-speaking Africans. PIG: Pygmies from the Central African Republic (1000genomes; Harteveld et al., 2002; Ribeiro et al., 2003).
